# Modification of Desulfurization Ash via Thermal CO_2_ Treatment: Oxidation Behavior, Carbonation Characteristics, and Mineral Transformation Mechanisms

**DOI:** 10.3390/molecules31172973

**Published:** 2026-08-25

**Authors:** Pengzhen Li, Ying Chen, Duo Li, Qian Wang, Hongyan Yan

**Affiliations:** 1 College of Energy and Environment, North China University of Science and Technology, Tangshan 063210, China; 2 School of Energy and Environment, Anhui University of Technology, Ma’anshan 243002, China

**Keywords:** desulfurization ash, CO_2_ uptake, carbonation, CaSO_3_, needle-like CaCO_3_, mechanism

## Abstract

Desulfurization ash (DA) is a kind of industrial solid waste generated during flue gas desulfurization. To overcome the limited utilization of reactive calcium-bearing constituents and the insufficient mineralogical stability of DA, this study proposes a thermal CO_2_ modification approach based on the synergistic coupling of oxidation and carbonation, and systematically investigates the associated evolution of mineral phases and microstructural features. The effects of reaction temperature, CO_2_ flow rate, and reaction time are evaluated using TG-DSC, XRD, FT-IR, SEM-EDS, particle size analysis, and carbon-sulfur analysis to elucidate the reaction behavior of DA under a CO_2_ atmosphere. The results demonstrate that reaction temperature is the dominant factor governing the modification process. During heating, CaSO_3_ is preferentially oxidized to CaSO_4_ under the CO_2_ atmosphere, thereby stabilizing the sulfur-bearing components. Subsequently, Ca(OH)_2_ undergoes carbonation to form CaCO_3_, simultaneously contributing to CO_2_ sequestration. Under the optimized conditions of 450 °C, a CO_2_ flow rate of 120 mL/min, and a reaction time of 60 min, the resulting CaCO_3_ exhibits a pronounced needle-like CaCO_3_ morphology, while the CO_2_ uptake reaches a maximum of 16.71%. These findings clarify the coupled oxidation–carbonation mechanism and mineral transformation behavior of DA during thermal CO_2_ modification, providing a theoretical basis and technical reference for the low-carbon utilization of industrial solid wastes.

## 1. Introduction

Rapid industrial development and the extensive consumption of coal-based energy have contributed substantially to atmospheric pollution in China. To mitigate these environmental impacts, considerable efforts have been devoted to developing advanced technologies and emission-control systems for reducing industrial air pollutants, particularly SO_2_ and NO_X_ [1,2,3,4,5]. Among these pollutants, SO_2_ remains a major atmospheric emission in China, with annual emissions reaching approximately 5.29 million tons [6,7]. To effectively reduce the adverse environmental impacts associated with SO_2_ emissions, flue gas desulfurization (FGD) has become an important approach for controlling air pollution from industrial sources. Among these available technologies, semi-dry and dry processes have been widely adopted in industrial applications because of their relatively low capital investment, reduced operating costs, and high operational adaptability [8,9,10]. However, the desulfurization process continuously generates substantial quantities of DA as a by-product; its subsequent valorization and safe disposal have become increasingly important challenges to the sustainable implementation of FGD technology. Available surveys indicate that semi-dry DA in China is still predominantly managed through stockpiling, which requires substantial land resources and may also pose additional risks of environmental contamination [11]. DA is primarily generated from calcium-based flue gas desulfurization processes, as the solid residue formed through reactions between lime-based sorbents and SO_2_ in flue gas. Its mineralogical composition generally includes CaSO_3_, CaSO_4_, Ca(OH)_2_, and CaO, together with minor amounts of Si, Al, and iron-containing oxides [12]. The high abundance of calcium-bearing constituents gives DA considerable potential for resource recovery and value-added utilization. Accordingly, it has been explored as a supplementary component in construction materials [13,14], a functional filler for products such as rubber and plastics [13,15], and a material for environmental remediation [16]. Nevertheless, the practical utilization of DA is hindered by its complex mineralogical composition, limited phase stability, and pronounced compositional variability. In particular, residual CaO and Ca(OH)_2_ exhibit poor volumetric stability and are susceptible to expansion upon contact with water, while the presence of CaSO_3_ may compromise the long-term stability of derived materials. These limitations significantly restrict the large-scale and high-value utilization of DA. Therefore, developing effective mineral regulation strategies to transform unstable phases, enhance structural stability, and facilitate resource recovery from this solid waste remains a critical challenge in the management and valorization of industrial solid wastes.

In recent years, CO_2_ mineralization using industrial solid wastes has emerged as a promising strategy that combines carbon sequestration with the beneficial utilization of waste resources [3,17]. This approach relies primarily on reactions between CO_2_ and alkaline oxides or hydroxides present in solid wastes, converting CO_2_ into thermodynamically stable carbonate minerals such as CaCO_3_ or MgCO_3_. In this way, long-term CO_2_ sequestration can be achieved while simultaneously improving the physicochemical properties of the solid waste. Previous studies have demonstrated that Ca- and Mg-rich industrial by-products, including steel slag [18,19], cement kiln dust [20], and fly ash [21], can undergo carbonation-induced mineral transformation. During this process, reactive phases such as CaO and Ca(OH)_2_ react with CO_2_ to form stable CaCO_3_, thereby reducing material alkalinity and enhancing volumetric stability. Compared with conventional disposal or treatment routes, CO_2_ mineralization offers the dual benefits of consuming substantial quantities of industrial solid waste and converting CO_2_ into stable mineral products, which has attracted increasing research interest in recent years. Given its high content of reactive calcium-bearing phases, DA therefore represents a promising feedstock for CO_2_ sequestration.

Building on these advantages, recent studies have explored CO_2_ mineralization as a means of modifying DA. By tailoring the reaction environment and operating parameters, the crystal morphology of CaSO_4_ and CaCO_3_ can be regulated, enabling the production of modified DA with targeted functional properties. Currently, modification strategies for DA can generally be classified into two routes: thermal and wet processes. Zhou et al. [22] and Baras et al. [23] employed hydrothermal treatment to tailor the phase composition and morphology of DA, promoting the preferential growth of calcium sulfate into whisker-like structures. Wang et al. [24] reported that calcium sulfate hemihydrate in CDA could be regulated using an ammonium citrate solution, allowing the modified product to be further utilized as an environmentally friendly rubber filler. Although wet modification processes generally achieve high oxidation efficiencies for calcium sulfite in DA, typically exceeding 98%, they often require additional operations such as solid–liquid separation and subsequent drying. These processing steps increase operating costs and present significant barriers to large-scale industrial implementation. Compared with wet modification, thermal treatment avoids additional operations such as solid–liquid separation and drying. Li et al. [25] and Yao et al. [26] investigated the oxidation behavior of mineral phases in DA under O_2_-containing atmospheres, with particular emphasis on the effects of reaction temperature, oxygen concentration, and holding time. Nevertheless, the coupled oxidation–carbonation behavior of DA during thermal treatment in a CO_2_ atmosphere has received limited attention, and the role of CO_2_ in governing these concurrent phase transformations remains insufficiently understood.

In order to solve the above problems, this study proposes a coupled oxidation–carbonation strategy for the thermal modification of DA under a CO_2_ atmosphere and investigates the associated reaction behavior and phase evolution. TG-DSC analysis is first employed to identify the mass and thermal evolution of DA during heating in CO_2_ and to distinguish the dominant reactions occurring within different temperature ranges. The effects of reaction temperature, CO_2_ flow rate, and holding time are then evaluated, while X-ray diffraction (XRD), Fourier-transform infrared spectroscopy (FT-IR), scanning electron microscopy (SEM), and carbon–sulfur analysis are used to track the transformation of key mineral phases, including Ca(OH)_2_, CaSO_3_, CaSO_4_, and CaCO_3_. In addition, the relationship between CO_2_ uptake and mineral phase evolution is examined to elucidate the coupling between oxidation and carbonation during CO_2_ treatment. The findings are expected to provide a new route for the high-value utilization of DA and a theoretical basis for integrating industrial solid-waste valorization with CO_2_ mitigation.

## 2. Materials and Methods

### 2.1. Materials

In this study, the experimental DA was purchased from an Environmental Protection Technology Co. in China. The inert gases used in the experiment were carbon dioxide (CO_2_, 99.99%) and argon gas (Ar, 99.99%). The chemical composition of the DA was determined by X-ray fluorescence (XRF), and the corresponding results are listed in Table 1. It can be observed that CaO and SO_3_ are the predominant chemical constituents of the DA, with contents of 59.50wt.% and 16.80wt.%, respectively. Furthermore, minor amounts of K_2_O, MgO, Na_2_O, and other trace oxides are also detected, indicating that the DA contains various minor inorganic components in addition to the dominant calcium-containing phases.

### 2.2. Methods

**Figure 1:**
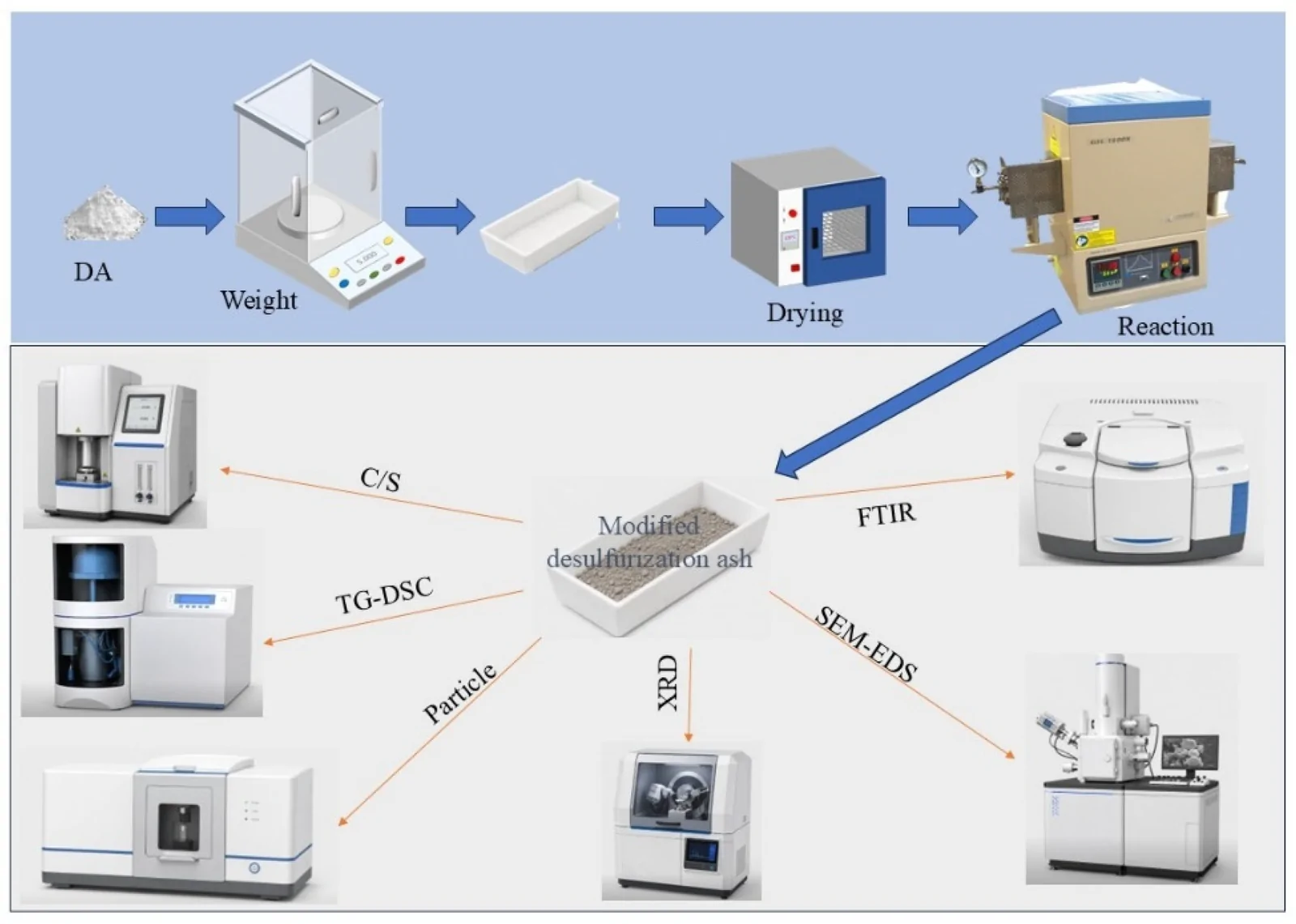
The schematic diagram of the experimental procedure.

A total of 3 g of DA sample is evenly spread in a porcelain boat and pre-dried in an oven at 120 °C for 4 h to remove crystalline water. Subsequently, the dried sample is transferred into a tubular furnace, where Ar gas is introduced at a flow rate of 150 mL/min for 5 min to eliminate residual air from the reaction system. After establishing a stable inert atmosphere, CO_2_ was introduced as the reaction gas, and the furnace temperature was increased from room temperature to the desired reaction temperature at a heating rate of 5 °C/min. After maintaining the sample at the target temperature for a predetermined period, the furnace was cooled to room temperature at a rate of 5 °C/min. The obtained products were then collected, sealed, and stored for subsequent characterization. The schematic diagram of the experimental procedure is shown in Figure 1.

In this study, all samples were stored in vacuum-sealed bags prior to characterization, and the storage environment was maintained under dry conditions. Moreover, the phases in the modified DA exhibited relatively high stability and were unlikely to undergo reactions with atmospheric components. Therefore, the influence of potential phase transformation caused by improper storage conditions can be considered negligible. The experimental design employed in this work is summarized in Table 2, where the subscripts in the sample labels represent different levels of the corresponding experimental parameters.

### 2.3. Characterizations

The phase composition of the DA and the chemical states of surface elements are characterized by X-ray diffraction (XRD, D/MAX 2500PC, Rigaku, Tokyo, Japan) and X-ray fluorescence spectroscopy (XRF, MagixPW2403, PANalytical B.V., Almelo, The Netherlands). The microstructures of the samples before and after modification are characterized using scanning electron microscopy (SEM, JEOL JSM-7800F (JEOL, Tokyo, Japan)/FEI Nova NanoSEM 400 (FEI, Hillsboro, OR, USA)) equipped with an energy-dispersive X-ray spectroscopy system (EDS, Oxford INCA Energy 350, Abingdon, UK), which is also used to analyze the elemental distribution in the samples. The TG-DSC of the samples during the modification process is analyzed by SetaramSetsysEvo (SETARAM Instrumentation, Caluire, France, SETSYS Evolution). Fourier-transform infrared spectroscopy (FT-IR, Thermo Scientific Nicolet iS20, Waltham, MA, USA) is used to analyze the DA before and after modification by identifying characteristic functional groups and chemical bonds in the solid phases, thereby providing insight into changes in molecular interactions, carbonation products such as carbonates, and hydrated phases. The particle size distribution of DA before and after modification was measured using a laser particle size analyzer (Malvern Panalytical Ltd., Mastersizer 3000, Malvern, UK), and the corresponding mass-specific surface area was calculated based on the particle size data. The carbon contents of the samples before and after modification were measured using a carbon-sulfur analyzer (CS844, LECO, St. Joseph, MI, USA).

### 2.4. CO_2_ Uptake

To determine the CO_2_ uptake, the carbon contents of the samples before and after the reaction were measured using a carbon-sulfur analyzer. For each measurement, 0.20 ± 0.03 g of sample is placed in a crucible and covered with approximately 1 g of T-type tin granules as a flux. The carbon content is then determined after combustion at 1050 °C for 5 s.

The carbon uptake during the reaction is calculated using Equation (1):

(1) $$\Delta C=C_{1}-C_{0}$$

where C_0_ represents the carbon content of the raw desulfurization ash, with a value of 4.083%, and C_1_ represents the carbon content of the DA after reaction.

The CO_2_ uptake is calculated using Equation (2):

(2) $$\vartheta ({\mathrm{CO}}_{2})=\frac{{\mathrm{MW}}_{{\mathrm{CO}}_{2}}}{{\mathrm{MW}}_{C}}\times \Delta C$$

where MW_C_ is the molar mass of carbon (12 g/mol) and MW_CO2_ is the molar mass of CO_2_ (44 g/mol).

## 3. Results and Discussion

### 3.1. Characterization of Raw Desulfurization Ash

**Figure 2:**
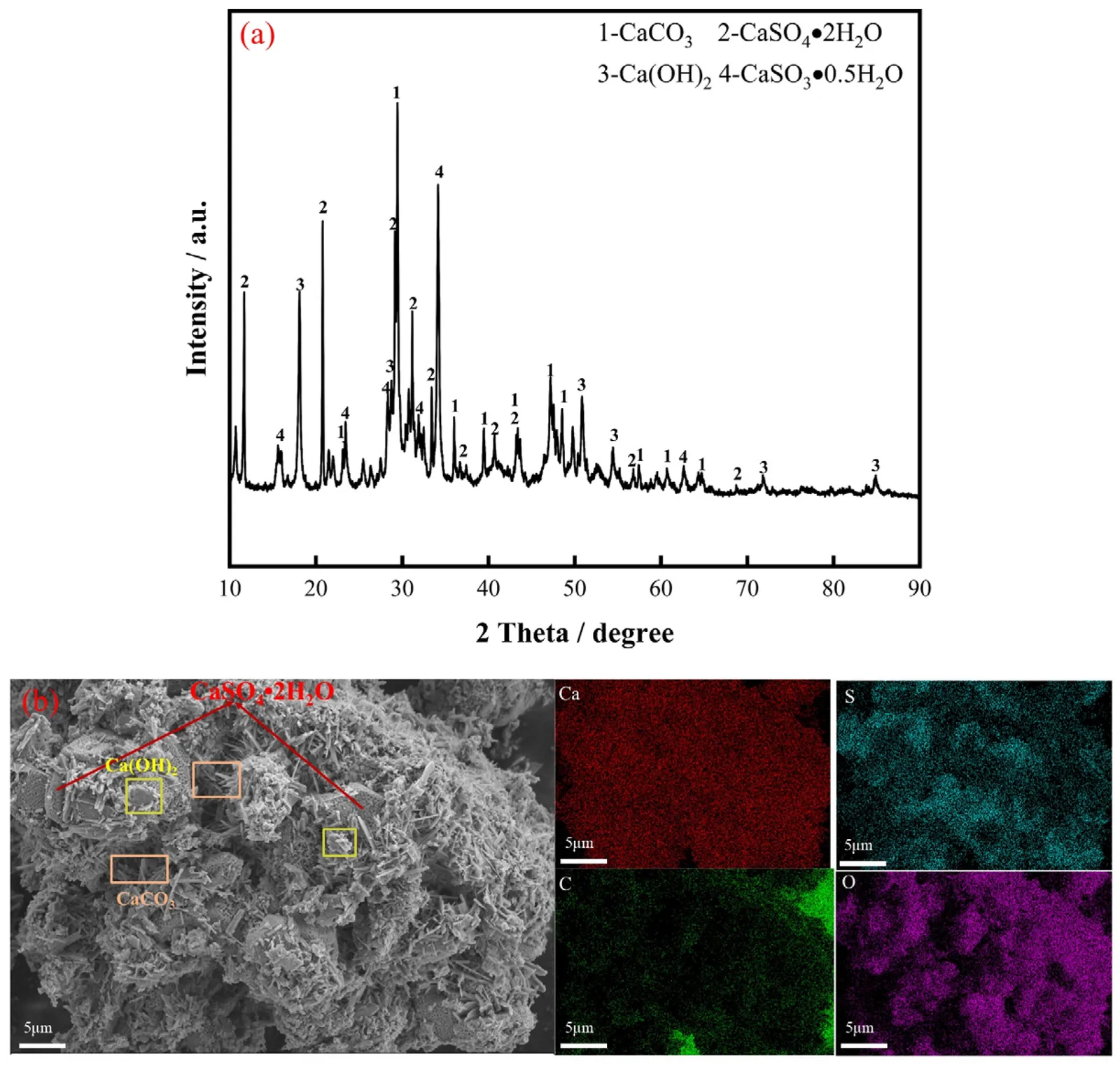
(**a**) XRD patterns of DA; (**b**) SEM-EDS analysis of DA.

The phase composition of the DA was characterized by X-ray diffraction (XRD), and the corresponding diffraction patterns are presented in Figure 2a. As illustrated, the pristine DA is primarily composed of CaCO_3_, CaSO_4_·2H_2_O, Ca(OH)_2_, and CaSO_3_·0.5H_2_O. The morphology of the original DA was further investigated by scanning electron microscopy (SEM), as shown in Figure 2b. The raw DA exhibits a relatively dense microstructure, where plate-like Ca(OH)_2_ and needle-like CaCO_3_ particles are observed to be attached to the surface of calcium sulfate particles. The aggregation and distribution characteristics of these phases may significantly affect the microstructural evolution and physicochemical properties of the DA during subsequent carbonation treatment.

### 3.2. TG-DSC Analysis

**Figure 3:**
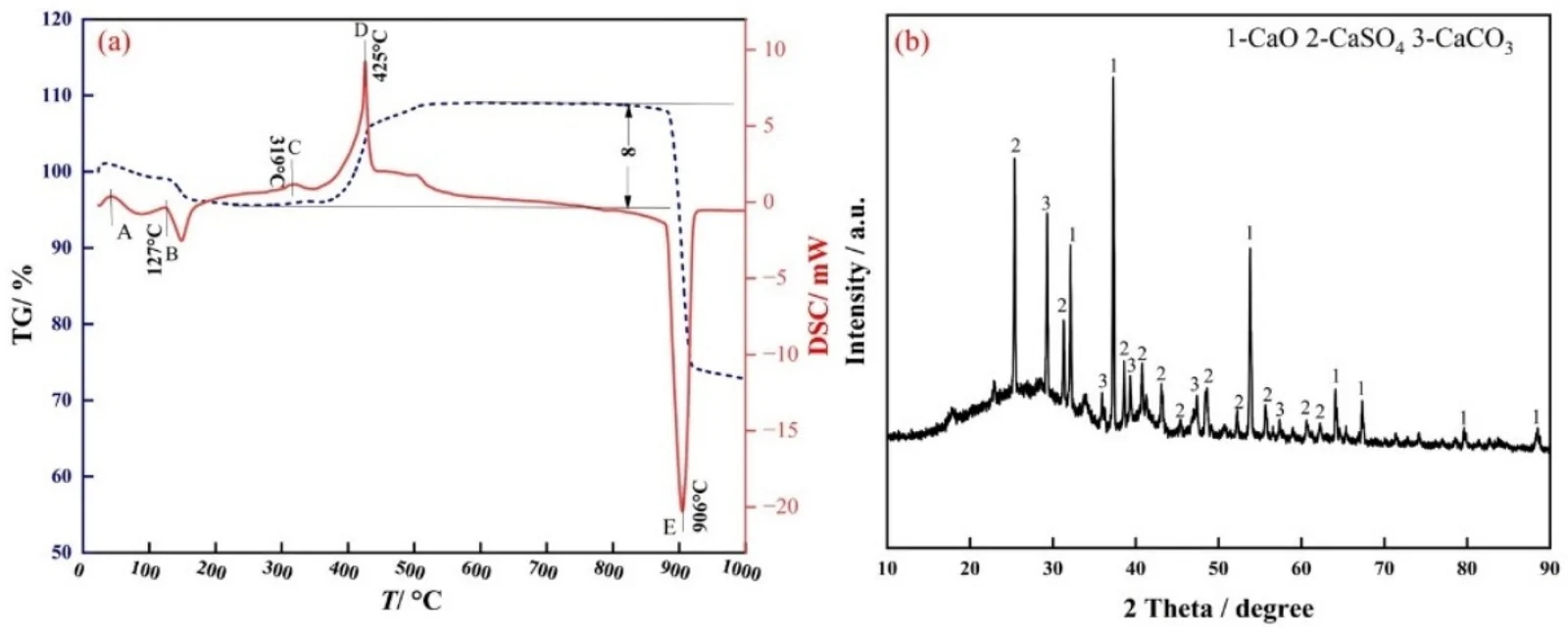
(**a**) TG-DSC curves of raw DA under a CO_2_ flow rate of 40 mL/min at a heating rate of 10 °C/min; (**b**) XRD patterns of the sample after the TG-DSC experiment.

Figure 3a presents the TG-DSC curves of DA heated from room temperature to 1000 °C at a heating rate of 10 °C/min under a CO_2_ flow of 40 mL/min. At the lower-temperature stage, peaks A and B are mainly associated with the release of crystalline water from CaSO_3_·0.5H_2_O and CaSO_4_·2H_2_O, respectively. With increasing temperature, peak C at approximately 319 °C is attributed to the oxidation of CaSO_3_ to CaSO_4_ under the CO_2_ atmosphere. Peak D, located at approximately 425 °C, is associated with the carbonation of Ca(OH)_2_. Within this temperature range, Ca(OH)_2_ in the DA undergoes a gas–solid reaction with CO_2_ to form stable CaCO_3_. At higher temperatures, CaSO_4_ and CaCO_3_ remain relatively stable until a pronounced endothermic reaction appears at approximately 906 °C (peak E). This event is mainly attributed to the thermal decomposition of CaCO_3_ [27], accompanied by a distinct mass loss. The XRD results in Figure 3b further show that no diffraction peaks corresponding to CaSO_3_ are detected after thermal treatment, indicating that the CaSO_3_ originally present in the DA is completely converted to CaSO_4_ during heating. Meanwhile, the CaO phase identified in the final product is primarily derived from the decomposition of CaCO_3_ at elevated temperatures.

### 3.3. The Influence of Different Factors on the Modification Effect of DA

#### 3.3.1. Temperature

**Figure 4:**
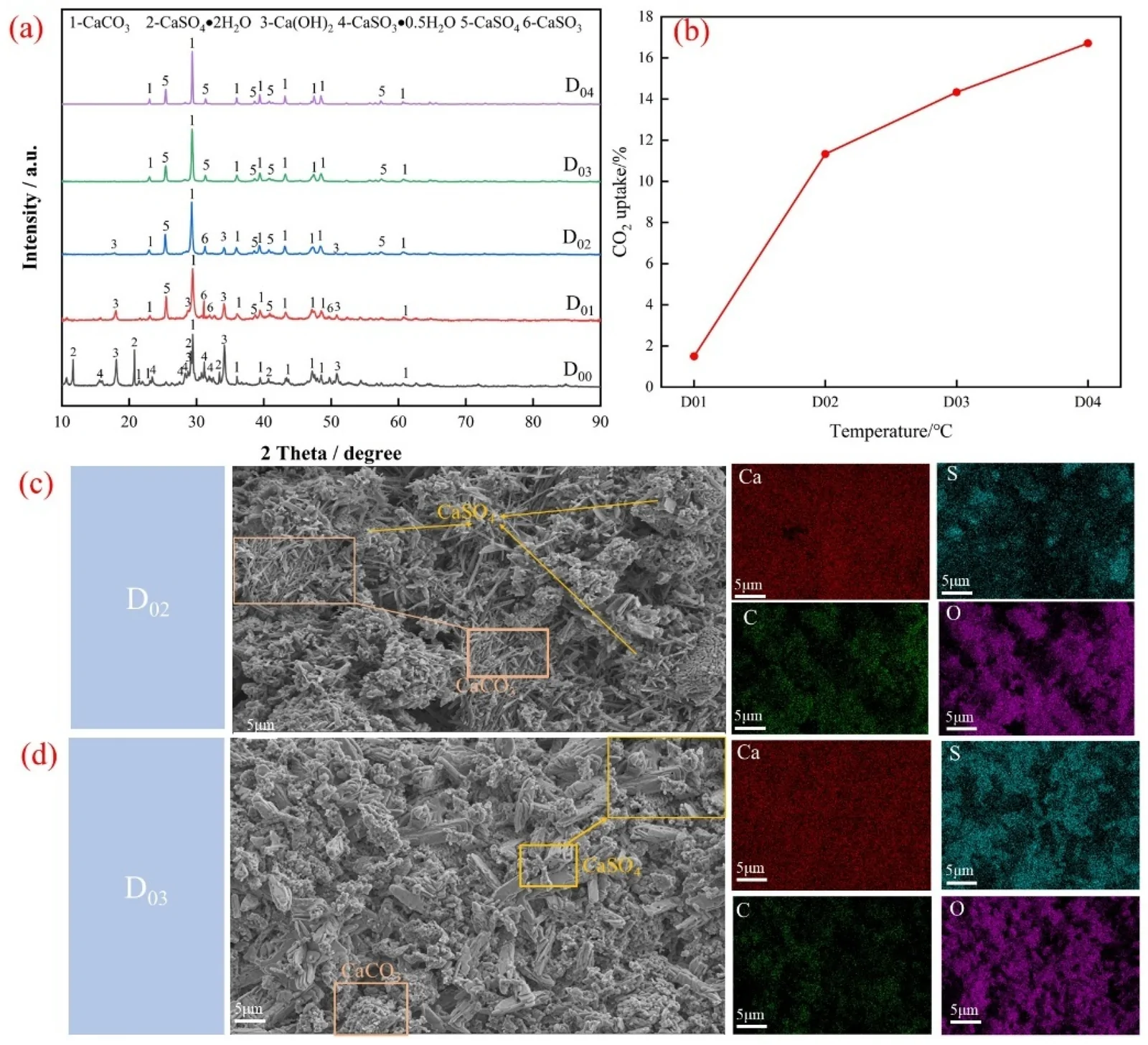
(**a**) XRD patterns of CO_2_-modified DA products obtained at different temperatures; (**b**) CO_2_ uptake of the DA block with different temperatures; (**c**) SEM-EDS analysis of 350 °C; (**d**) SEM-EDS analysis of 370 °C.

From the analysis of Section 3.2, it can be seen that before 310 °C, the DA system mainly undergoes the process of crystal water removal, and no obvious CO_2_ fixation reaction has occurred. Figure 4 further illustrates the effect of reaction temperature on the modification of DA at a fixed reaction time of 60 min and a CO_2_ flow rate of 120 mL/min. The XRD patterns show that, at temperatures below 350 °C, distinct diffraction peaks of Ca(OH)_2_ and CaSO_3_ remain in the products, indicating that both the oxidative conversion of CaSO_3_ and the carbonation of Ca(OH)_2_ are incomplete within this temperature range. At relatively low temperatures, the oxidation kinetics of CaSO_3_ under the CO_2_ atmosphere are limited, leaving part of the CaSO_3_ unconverted to CaSO_4_. Meanwhile, the carbonation of Ca(OH)_2_ to CaCO_3_ is constrained by both the reaction kinetics and CO_2_ diffusion, resulting in a considerable amount of unreacted calcium-bearing phases remaining in the system. As the reaction temperature increases above 350 °C, the diffraction peaks of CaSO_3_ and Ca(OH)_2_ gradually weaken, whereas the characteristic peaks of CaCO_3_ become progressively more intense. CaSO_3_ is further oxidized to CaSO_4_ under the CO_2_ atmosphere, consistent with the oxidation-related exothermic peak observed at approximately 319 °C in the TG-DSC analysis. This behavior indicates that the residual CaSO_3_ in DA retains high oxidative reactivity and can undergo further oxidation in a CO_2_-containing atmosphere. Meanwhile, the progressive increase in CaCO_3_ peak intensity indicates continuous carbonation of Ca(OH)_2_ by CO_2_, thereby contributing to CO_2_ sequestration. At 370 °C, the diffraction peaks of CaSO_3_ and Ca(OH)_2_ nearly disappear, and the products are predominantly composed of CaSO_4_ and CaCO_3_. Further increases in temperature produce only minor changes in the XRD patterns, suggesting that the phase transformation reactions are essentially complete. Figure 4b shows the variation in CO_2_ uptake with reaction temperature. At relatively low temperatures, CO_2_ uptake increases only slightly because of the sluggish carbonation kinetics. Upon increasing the temperature to 370 °C, CO_2_ uptake rises progressively as the reaction proceeds, indicating a marked enhancement in the CO_2_ sequestration capacity of the system.

Figure 4c,d further present the SEM-EDS results of the products obtained at 350 and 370 °C under a fixed reaction time of 60 min and a CO_2_ flow rate of 120 mL/min. At 350 °C, CaSO_4_ retains morphological features similar to those observed in the original DA, appearing predominantly as irregular block-like particles with porous surfaces. This observation suggests that no pronounced morphological reconstruction of the CaSO_4_ phase has occurred at this temperature. Meanwhile, abundant needle-like CaCO_3_ crystals are observed on the particle surfaces. When the reaction temperature is increased to 370 °C, the morphology of the CaSO_4_ particles changes markedly, with the original porous block-like structure gradually evolving into an irregular layered morphology.

#### 3.3.2. CO_2_ Flow Rate

**Figure 5:**
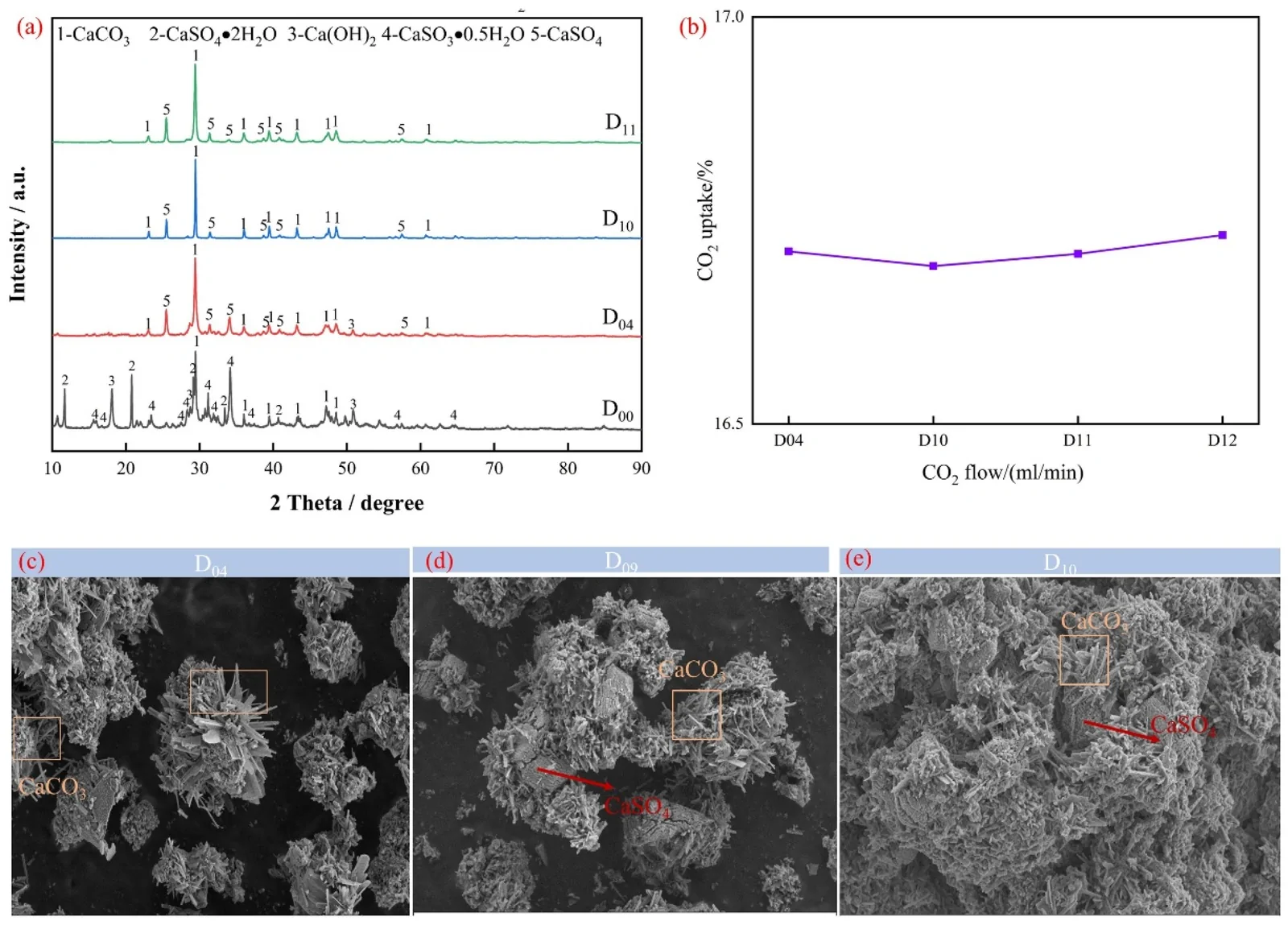
(**a**) XRD patterns of CO_2_-modified DA products obtained at different CO_2_ flow rates; (**b**) CO_2_ uptake of the DA block with different CO_2_ flow rates; (**c**) SEM-EDS analysis of 120 mL/min CO_2_ flow rate; (**d**) SEM-EDS analysis of 150 mL/min CO_2_ flow rate; (**e**) SEM-EDS analysis of 180 mL/min CO_2_ flow rate.

The change in CO_2_ flow rate will affect the supply of CO_2_ in the reaction system and the gas–solid contact process, which may affect the modification process of DA [28]. Figure 5 examines the influence of CO_2_ flow rate on the modification process at 450 °C with a fixed reaction time of 60 min. As shown by the XRD patterns in Figure 5a, increasing the CO_2_ flow rate produces no appreciable change in the phase composition of the products, which are predominantly composed of CaCO_3_ and CaSO_4_. This result suggests that, under these reaction conditions, increasing the CO_2_ supply does not further promote the transformation of the reactive calcium-bearing phases in DA. This trend is further supported by the CO_2_ uptake results presented in Figure 5b, where only minor changes in CO_2_ uptake are observed as the CO_2_ flow rate increases.

Figure 5c–e present the SEM images of the products obtained at different CO_2_ flow rates. The CO_2_ flow rate exerts a pronounced influence on the morphological evolution of CaCO_3_ crystals. As the CO_2_ flow rate increases, the size of the needle-like CaCO_3_ crystals gradually decreases. At a CO_2_ flow rate of 120 mL/min, the products mainly contain short clustered CaCO_3_ crystals together with needle-like CaCO_3_ exhibiting a relatively high aspect ratio, indicating that an adequate CO_2_ supply favors the rapid interaction between Ca^2+^ and CO_3_^2−^ and subsequent crystal growth. With a further increase in CO_2_ flow rate, the enhanced CO_2_ availability accelerates carbonate formation, while excessive nucleation restricts subsequent crystal growth [29,30]. Consequently, the short clustered CaCO_3_ crystals gradually decrease in abundance and evolve toward smaller needle-like CaCO_3_ crystals with lower aspect ratios. Meanwhile, the newly formed needle-like CaCO_3_ crystals progressively cover the surfaces of CaSO_4_ particles, producing a coating structure. Combined with the XRD results, these observations indicate that although increasing the CO_2_ flow rate affects CaCO_3_ crystallization and morphology, it does not cause an appreciable change in the overall phase composition. This suggests that, at 450 °C and a CO_2_ flow rate of 120 mL/min, the CO_2_ supply is already sufficient for the reaction. Further increases in flow rate therefore mainly influence the nucleation and morphological development of CaCO_3_ rather than substantially enhancing the overall CO_2_ uptake.

#### 3.3.3. Reaction Time

**Figure 6:**
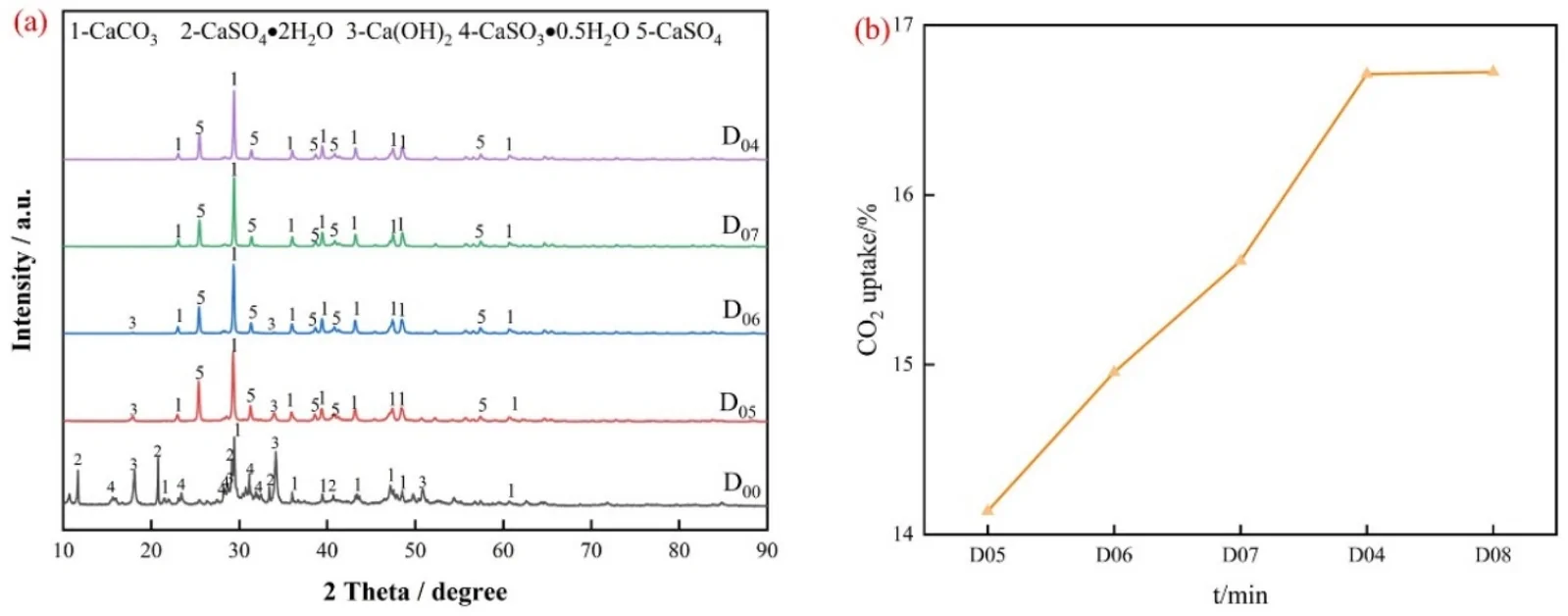
(**a**) XRD patterns of CO_2_-modified DA products obtained at different times; (**b**) CO_2_ uptake of the DA block with different reaction times.

Figure 6 illustrates the effect of reaction time on the modification of DA at 450 °C under a constant CO_2_ flow rate. Figure 6a presents the XRD patterns of the products obtained after holding times of 5, 10, 20, and 60 min. After the reaction time of 5 min, the diffraction peaks corresponding to CaSO_3_ in the original DA have nearly disappeared, indicating that CaSO_3_ undergoes rapid oxidative conversion under the CO_2_ atmosphere. When the holding time is extended to 10 min, the characteristic diffraction peaks of CaCO_3_ become progressively more intense. After 20 min, the products are predominantly composed of CaCO_3_ and CaSO_4_, suggesting that the highly reactive calcium-bearing phases in the DA, including Ca(OH)_2_ and CaSO_3_, have been largely transformed. Further extending the holding time to 60 min produces no appreciable change in the XRD patterns, indicating that the major mineral phases have essentially stabilized. Figure 6b further shows the variation in CO_2_ uptake with holding time. As the holding time increases from 5 to 60 min, CO_2_ uptake increases continuously, accompanied by the progressive conversion of reactive calcium-bearing phases into stable CaCO_3_. Beyond 60 min, however, the CO_2_ uptake approaches a plateau, and prolonging the reaction time produces little additional increase in CO_2_ sequestration. This behavior indicates that the overall conversion rate gradually decreases at the later stage of the reaction. This trend may be associated with CO_2_ diffusion within the particles and mass transfer at the reaction interface.

#### 3.3.4. Gas Type

**Figure 7:**
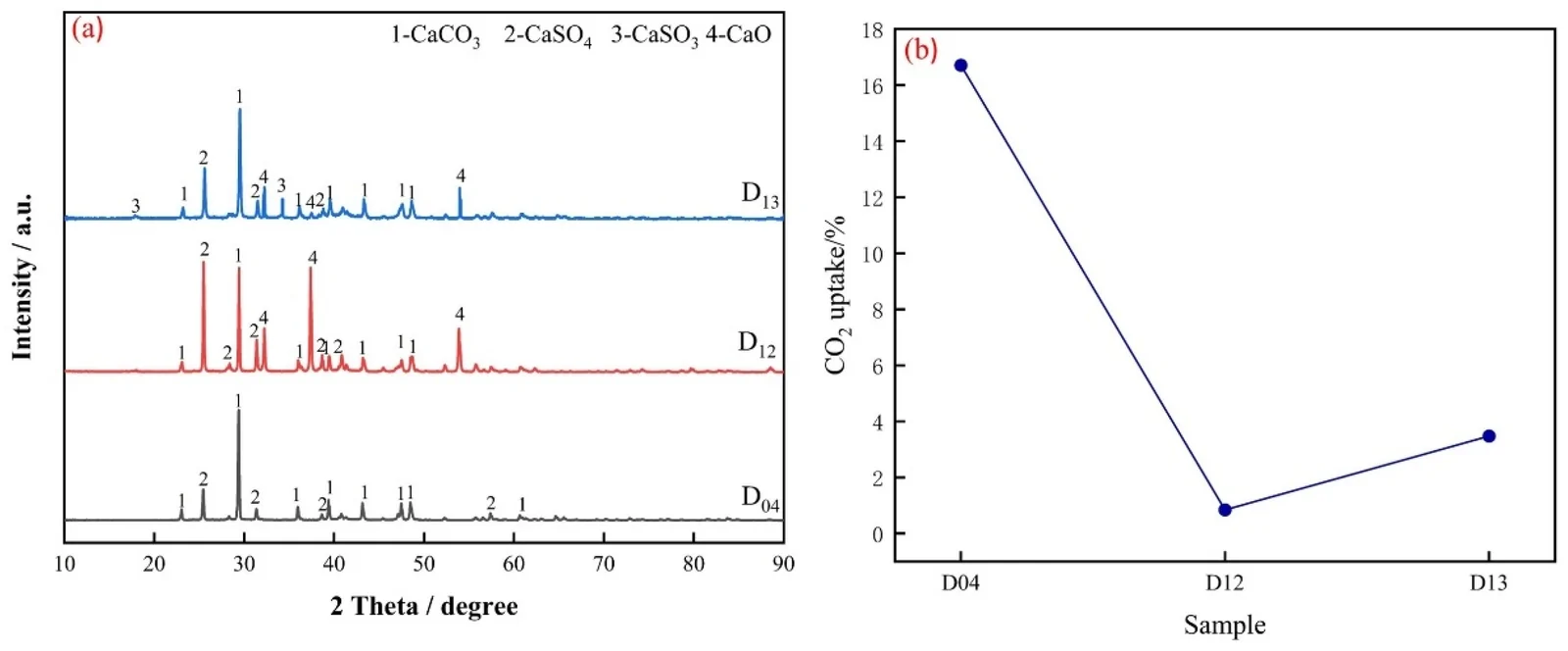
(**a**) XRD patterns of DA modified under different gas atmospheres; (**b**) CO_2_ uptake of DA modified under different gas atmospheres.

Figure 7 illustrates the effects of different gas atmospheres on the modification behavior of DA at 450 °C with a gas flow rate of 120 mL/min. As shown in Figure 7a, CaSO_4_ and CaCO_3_ are identified as the major crystalline phases after treatment under an air atmosphere. The presence of O_2_ in air promoted the oxidation of CaSO_3_ to CaSO_4_, resulting in CaSO_4_ becoming the predominant sulfate phase in the modified sample. Meanwhile, because no external CO_2_ is supplied under the air atmosphere, the formation of CaCO_3_ is mainly associated with the carbonate species originally present in DA and the limited carbonation reactions occurring during treatment. Consequently, the diffraction peak intensities of CaSO_4_ and CaCO_3_ are relatively comparable. In contrast, Ar is chemically inert under the experimental conditions and does not participate appreciably in reactions with the active components of DA. Therefore, the thermal decomposition of Ca(OH)_2_ is the primary reaction occurring under the Ar atmosphere, while no obvious oxidation conversion of CaSO_3_ is observed. These results indicate that Ar mainly serves as a protective atmosphere during the modification process and has a limited direct effect on the phase transformation of DA. This finding further confirms that the Ar component in the Ar and CO_2_ mixed atmosphere does not substantially interfere with the reaction between CO_2_ and DA.

Figure 7b compares the CO_2_ uptake of DA modified under different gas atmospheres. The sample treated under a CO_2_ atmosphere exhibited the highest CO_2_ uptake, which can be mainly attributed to the continuous supply of CO_2_ and its subsequent carbonation with the reactive calcium-containing components in DA. In particular, Ca(OH)_2_ possesses relatively high reactivity toward CO_2_, thereby facilitating CO_2_ absorption and fixation and consequently increasing the carbon content of the modified sample. In contrast, neither Ar nor air provides an external source of CO_2_, resulting in a substantially lower CO_2_ uptake than that obtained under the CO_2_ atmosphere. Furthermore, under the air atmosphere, O_2_ promoted the conversion of CaSO_3_ to CaSO_4_, which further reduced the amount of reactive calcium available for carbonation and thus contributed to the relatively low CO_2_ uptake.

### 3.4. Particle Analysis

**Figure 8:**
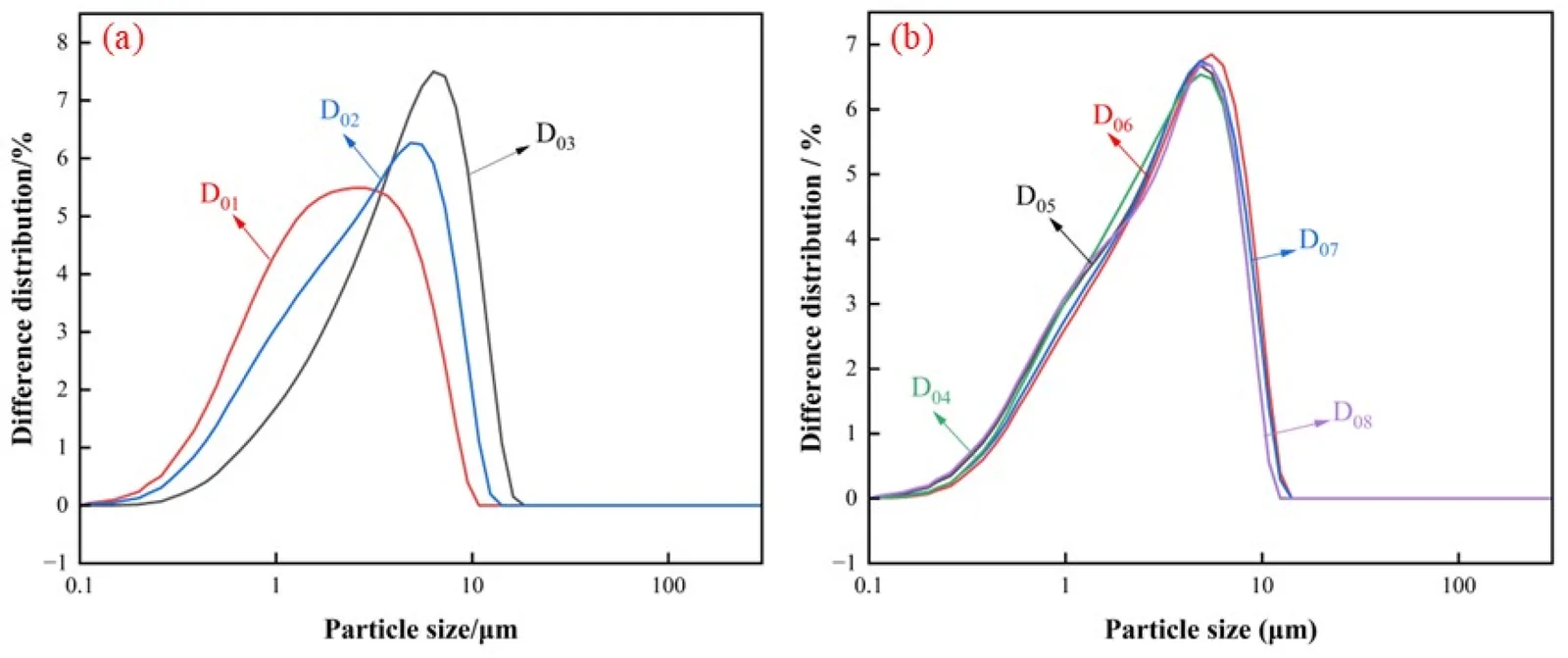
(**a**) Particle size distribution curves of different temperatures; (**b**) particle size distribution curves of different times.

Figure 8 illustrates the evolution of particle size distribution during the modification of DA. All samples exhibit unimodal particle size distributions, with most particles falling within the range of 0.1–10 μm. As the modification conditions change, the particle size distribution progressively shifts toward larger sizes, with the peak position increasing from approximately 2 to 7 μm, indicating an overall increase in particle size during the modification process. This behavior may be associated with the formation of CaCO_3_ during CO_2_-induced carbonation.

To further characterize the changes in the particle characteristics of DA during CO_2_ modification, the weight-specific surface area and mean particle size of the samples obtained under different reaction conditions were analyzed, as summarized in Table 3. The raw DA exhibits a weight-specific surface area of 3.601 m^2^/kg and a mean particle size of 5.83 μm. Both parameters vary with reaction temperature. At 310 °C, the weight-specific surface area decreases to 2.408 m^2^/kg, while the mean particle size is 4.72 μm. When the temperature increases to 350 °C, the weight-specific surface area increases to 5.029 m^2^/kg, accompanied by a decrease in the mean particle size to 2.39 μm. At 370 °C, the weight-specific surface area decreases to 3.812 m^2^/kg, whereas the mean particle size increases to 3.31 μm. These results demonstrate that the particle size characteristics and weight-specific surface area of DA vary appreciably with reaction temperature, corresponding to the changes in mineral composition and microstructure observed during the modification process. At 450 °C, the weight-specific surface area of the samples obtained at different reaction times ranges from 2.973 to 3.896 m^2^/kg, while the mean particle size ranges from 3.24 to 4.43 μm, indicating relatively limited overall variation. Further analysis of the effect of reaction time shows that both the weight-specific surface area and mean particle size change with increasing reaction time. After 5 min, the sample exhibits a weight-specific surface area of 3.452 m^2^/kg and a mean particle size of 3.33 μm. When the reaction time is extended to 10 min, the weight-specific surface area decreases to 3.051 m^2^/kg, while the mean particle size increases to 4.38 μm. With a further increase in reaction time to 120 min, both parameters continue to fluctuate to some extent, although the magnitude of the changes gradually decreases. Overall, the particle size distribution, weight-specific surface area, and mean particle size results demonstrate that the CO_2_ modification conditions affect the particle characteristics of DA, while these parameters tend to become relatively stable at longer reaction times.

### 3.5. FT-IR Analysis

**Figure 9:**
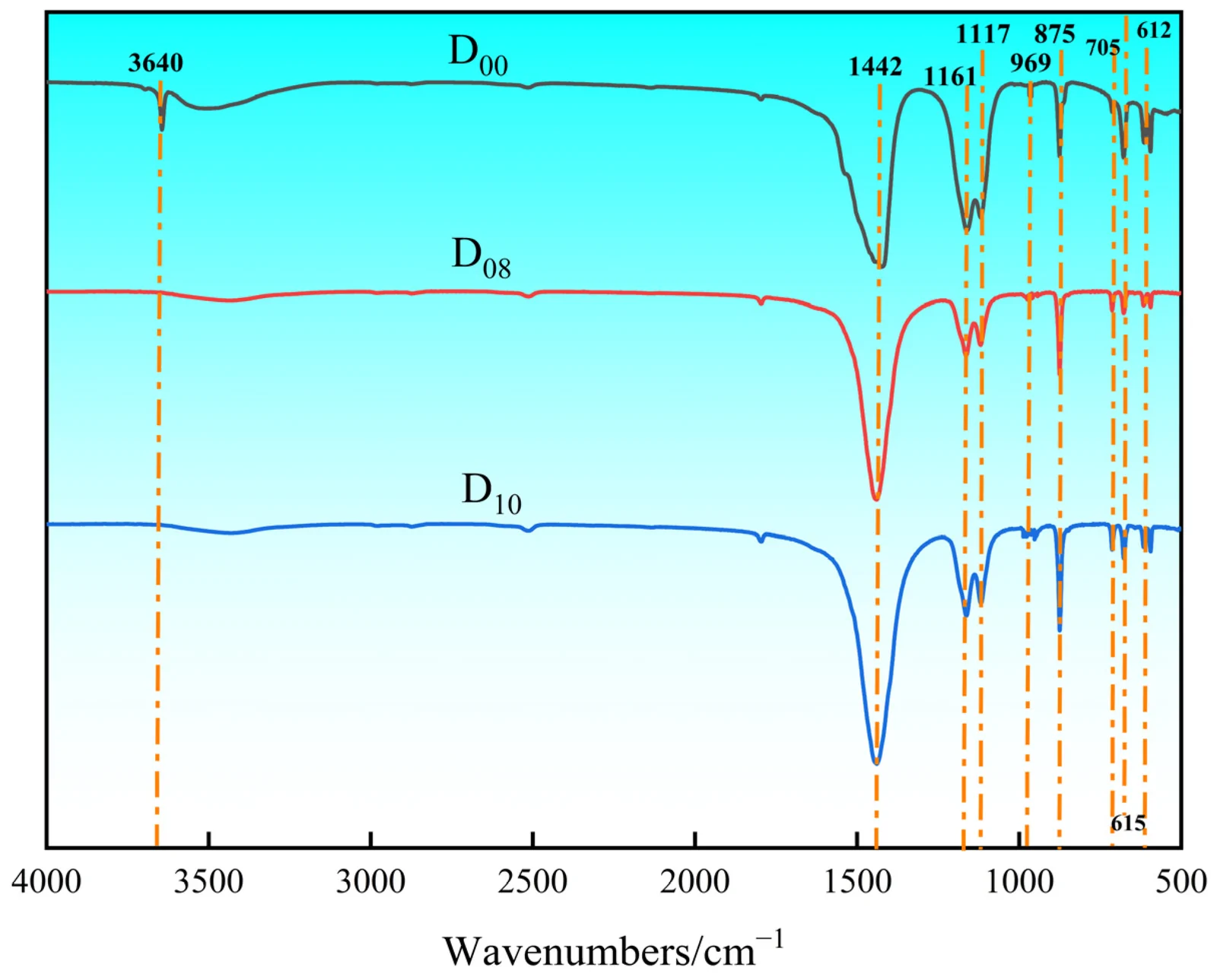
Results of FT-IR analysis of DA compacts of different CO_2_ conditions.

Figure 9 presents the Fourier-transform infrared (FT-IR) spectra of the raw DA and the samples modified under different CO_2_ conditions. For the untreated sample, the absorption band at approximately 3640 cm^−1^ is assigned to the O-H stretching vibration of hydroxyl groups in Ca(OH)_2_, indicating the presence of a considerable amount of residual Ca(OH)_2_ in the raw DA [31]. After treatment at 450 °C, the intensity of this band decreases markedly, suggesting progressive consumption of hydroxyl-bearing Ca(OH)_2_ through carbonation with CO_2_ to form CaCO_3_. This observation is consistent with the XRD results, which show a gradual weakening and eventual disappearance of the Ca(OH)_2_ diffraction peaks accompanied by an increase in the intensity of the CaCO_3_ peaks. The strong absorption band at approximately 1442 cm^−1^ is attributed to the asymmetric C-O stretching vibration of CO_3_^2−^. Its progressive enhancement after CO_2_ treatment indicates the conversion of CO_2_ into stable carbonate phases. Additional carbonate-related bands are observed at approximately 875 and 705 cm^−1^. Together with the observed needle-like morphology in the SEM images, these spectral features support the formation of needle-like CaCO_3_. The absorption band near 969 cm^−1^ is assigned to vibrations of SO_3_^2−^ groups, and its gradual attenuation with reaction progress indicates the continuous consumption of CaSO_3_ [23]. In contrast, the band near 612 cm^−1^, associated with vibrational modes of SO_4_^2−^, becomes progressively more intense, indicating the oxidative conversion of calcium sulfite into the more stable calcium sulfate phase. These FT-IR observations are consistent with the XRD, SEM-EDS, and TG-DSC results. Overall, the results demonstrate pronounced mineral-phase evolution during CO_2_ modification of DA. Reactive phases such as Ca(OH)_2_ and CaSO_3_ are progressively consumed, accompanied by the formation of needle-like CaCO_3_ and the oxidative transformation of CaSO_3_ into CaSO_4_. These coupled transformations contribute simultaneously to CO_2_ mineral sequestration and the stabilization of the DA.

### 3.6. Reaction Mechanism

#### 3.6.1. Thermomechanical Analysis

To elucidate the effect of the CO_2_ atmosphere on the oxidative conversion of CaSO_3_ at 450 °C, the influence of gas partial pressure on the Gibbs free energy of the reaction was analyzed. For reaction (3), since both CaSO_3_ and CaSO_4_ are solid phases, their activities can be approximated as unity. Therefore, the actual Gibbs free energy of the reaction can be expressed by Equation (4). At 450 °C, the equilibrium condition of this reaction is determined by the P_CO_/P_CO2_ ratio. In this experiment, CO_2_ is continuously supplied to the reaction system, while the generated CO is continuously removed from the reaction zone by the flowing gas, thereby suppressing the local accumulation of CO on the solid surface and maintaining a relatively high P_CO2_/P_CO_ ratio. Consequently, the continuous supply of CO_2_ can regulate the gas-phase chemical potential and the partial-pressure relationship between reactants and products, thereby enhancing the thermodynamic driving force for the conversion of CaSO_3_ to CaSO_4_. These results demonstrate, from a thermodynamic perspective, the feasibility of the experimental design adopted in this study.

(3) $${\mathrm{CaSO}}_{3}+{\mathrm{CO}}_{2}={\mathrm{CaSO}}_{4}+\mathrm{CO}$$

(4) $$\Delta G=\Delta G^{\theta }+\mathrm{RT}\ln(\frac{P_{\mathrm{CO}}}{P_{{\mathrm{CO}}_{2}}})$$

where ΔG is the Gibbs free energy change under the actual reaction conditions, kJ/mol; ΔG^θ^ is the standard Gibbs free energy change in the reaction, kJ/mol; R is the universal gas constant, 8.314 J/(mol·K^−1^); T is the absolute temperature, K; and P_CO_ and P_CO2_ are the partial pressures of CO and CO_2_ in the reaction system, respectively.

#### 3.6.2. Reaction Procress

According to the TG-DSC results presented in Section 3.1, the thermal reaction of DA under a CO_2_ atmosphere proceeds through several distinct stages. Within the lower-temperature range of 100–200 °C, dehydration of the hydrated mineral phases predominates. The crystalline water in CaSO_3_·0.5H_2_O and CaSO_4_·2H_2_O is progressively released, yielding anhydrous CaSO_3_ and CaSO_4_, respectively. No appreciable CO_2_ sequestration occurs during this stage, and the corresponding reactions are described by reactions (5) and (6). As the temperature increases, pronounced mineral-phase transformations begin to occur. Two endothermic peaks appear on the DSC curve at approximately 319 and 425 °C, corresponding to the oxidative conversion of CaSO_3_ to CaSO_4_ and the carbonation of Ca(OH)_2_ by CO_2_, respectively, as represented by reactions (3) and (7). These results indicate that, during heating, the reaction pathway of DA evolves sequentially from dehydration to oxidation and subsequently to carbonation.

**Figure 10:**
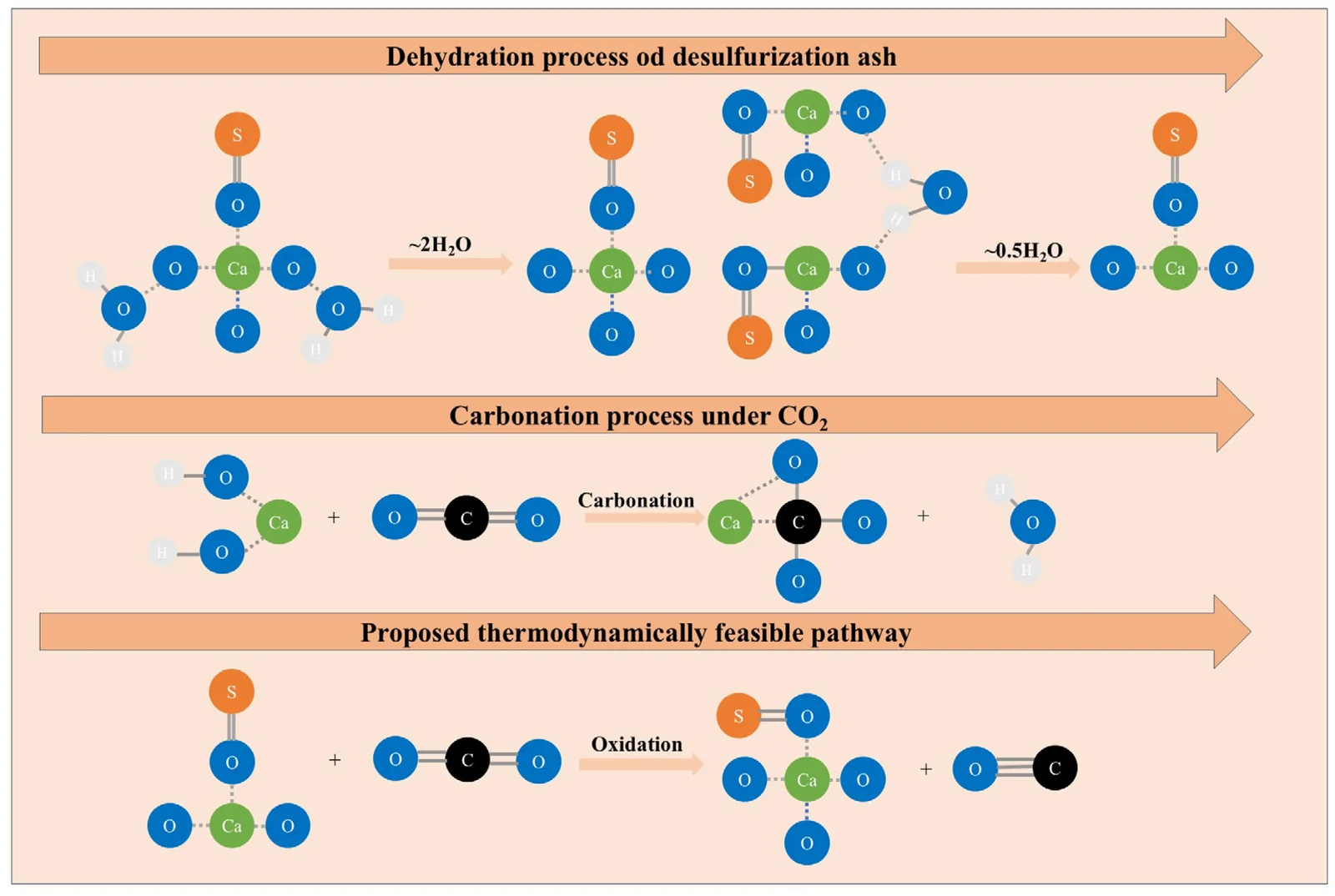
Reaction mechanism diagram.

Further combined with the XRD results under different reaction times in Section 3.3.3, it can be found that after a reaction time of only 5 min, the characteristic diffraction peaks of CaSO_3_ in the raw material have nearly disappeared, while the relative abundance of CaSO_4_ increases markedly; in contrast, distinct Ca(OH)_2_ diffraction peaks remain detectable. These observations indicate that, under the conditions employed in this study, the oxidative conversion of calcium sulfite occurs preferentially, whereas the carbonation of Ca(OH)_2_ proceeds subsequently and continues with increasing reaction time. As the reaction progresses, Ca(OH)_2_ is gradually consumed and converted into CaCO_3_. Therefore, the thermal modification of DF under CO_2_ is not a simple carbonation process but instead follows a sequential pathway involving dehydration of hydrated phases, oxidation of CaSO_3_, and carbonation of Ca(OH)_2_. The corresponding reaction pathway and mineral-phase evolution are schematically illustrated in Figure 10.

(5) $${\mathrm{CaSO}}_{3}\cdot 0.5H_{2}O={\mathrm{CaSO}}_{3}+0.5H_{2}O$$

(6) $${\mathrm{CaSO}}_{4}\cdot 2H_{2}O={\mathrm{CaSO}}_{4}+2H_{2}O$$

(7) $${\mathrm{Ca}(\mathrm{OH})}_{2}+{\mathrm{CO}}_{2}={\mathrm{CaCO}}_{3}+H_{2}O$$

## 4. Conclusions

In this study, DA was thermally modified under a CO_2_ atmosphere, and the oxidative conversion of CaSO_3_ and the carbonation behavior of Ca(OH)_2_ during the modification process were investigated. The effects of reaction temperature, CO_2_ flow rate, and reaction time on mineral-phase evolution, CO_2_ uptake, and microstructural changes were also evaluated. The main conclusions are summarized as follows:

(1) Reaction temperature is the dominant factor governing the modification process, while CO_2_ flow rate and reaction time also play important roles. Under a CO_2_ atmosphere, CaSO_3_ and Ca(OH)_2_ in the DA undergo oxidative conversion and carbonation, forming CaSO_4_ and CaCO_3_, respectively. At 450 °C, the modified product exhibits the highest CO_2_ uptake, reaching 16.71%.
(2) The modification process alters the mineral-phase composition of DA and enables regulation of the crystalline characteristics of the resulting phases. Under the conditions of 450 °C, a CO_2_ flow rate of 120 mL/min, and a reaction time of 60 min, SEM observations reveal that CaCO_3_ exhibits a distinct needle-like CaCO_3_ morphology. In addition, the interconnection of newly formed CaCO_3_ particles during modification leads to a moderate increase in particle size, further demonstrating the influence of mineral transformation on the structural evolution of DA particles.
(3) This study elucidates the coupled oxidation–carbonation mechanism of DA under a CO_2_ atmosphere, enabling the simultaneous transformation of unstable sulfur-bearing phases and reactive calcium-bearing components. Compared with conventional wet modification processes, this approach avoids complicated processing steps and provides a promising route for the high-value utilization of DA and the integration of industrial solid-waste valorization with CO_2_ sequestration.

## Figures and Tables

**Table 1:** Chemical composition of DA materials.

DA	Chemical Composition (%)						
	CaO	SO_3_	K_2_O	MgO	Na_2_O	Fe_2_O_3_	Others
	59.50	16.80	0.913	0.732	3.373	0.263	21.42

Data are from a single experiment.

**Table 2:** Experimental design for CO_2_ oxidation modification of DA.

Groups	Sample Name	Temperature (°C)	Gas Type	Gas Flow Rate (mL/min)	Time (min)
1	D_00_	0	CO_2_	120	60
2	D_01_	310	CO_2_	120	60
3	D_02_	350	CO_2_	120	60
4	D_03_	370	CO_2_	120	60
5	D_04_	450	CO_2_	120	60
6	D_05_	450	CO_2_	120	5
7	D_06_	450	CO_2_	120	10
8	D_07_	450	CO_2_	120	20
9	D_08_	450	CO_2_	120	120
10	D_09_	450	CO_2_	150	60
11	D_10_	450	CO_2_	180	60
12	D_11_	450	CO_2_	250	60
13	D_12_	450	Ar	120	60
14	D_13_	450	Air	120	60

**Table 3:** Physicochemical properties of modified DA.

Sample Name	Surface Area (m^2^/kg)	Average Particle Size (μm)
D_00_	3.601	5.83
D_01_	2.408	4.72
D_02_	5.029	2.39
D_03_	3.812	3.31
D_04_	2.973	4.43
D_05_	3.452	3.33
D_06_	3.051	4.38
D_07_	3.896	3.24
D_08_	4.180	3.04
D_09_	3.281	3.68
D_10_	3.489	3.51
D_11_	3.664	3.26

Data are presented as the mean of three independent experiments.

## Data Availability

The original contributions presented in this study are included in the article. Further inquiries can be directed to the corresponding author.
